# Evaluation of decompressive craniectomy in mice after severe traumatic brain injury

**DOI:** 10.3389/fneur.2022.898813

**Published:** 2022-07-26

**Authors:** Yuheng Liu, Xuanhui Liu, Zhijuan Chen, Yuanzhi Wang, Jing Li, Junjie Gong, Anqi He, Mingyu Zhao, Chen Yang, Weidong Yang, Zengguang Wang

**Affiliations:** ^1^Department of Neurosurgery, Tianjin Medical University General Hospital, Tianjin, China; ^2^Tianjin Neurological Institute, Key Laboratory of Post-Neuroinjury Neuro-repair and Regeneration in Central Nervous System, Ministry of Education and Tianjin, Tianjin, China; ^3^Department of Pharmacy, Tianjin Medical University General Hospital, Tianjin, China

**Keywords:** traumatic brain injury, decompressive craniectomy, controlled cortical impact (CCI), animal model, severe traumatic brain injury (sTBI)

## Abstract

Decompressive craniectomy (DC) is of great significance for relieving acute intracranial hypertension and saving lives after traumatic brain injury (TBI). In this study, a severe TBI mouse model was created using controlled cortical impact (CCI), and a surgical model of DC was established. Furthermore, a series of neurological function assessments were performed to better understand the pathophysiological changes after DC. In this study, mice were randomly allocated into three groups, namely, CCI group, CCI+DC group, and Sham group. The mice in the CCI and CCI+DC groups received CCI after opening a bone window, and after brain injury, immediately returned the bone window to simulate skull condition after a TBI. The CCI+DC group underwent DC and contused tissue removal 6 h after CCI. The mice in the CCI group underwent the same anesthesia process; however, no further treatment of the bone window and trauma was performed. The mice in the Sham group underwent anesthesia and the process of opening the skin and bone window, but not in the CCI group. Changes in Modified Neurological Severity Score, rotarod performance, Morris water maze, intracranial pressure (ICP), cerebral blood flow (CBF), brain edema, blood–brain barrier (BBB), inflammatory factors, neuronal apoptosis, and glial cell expression were evaluated. Compared with the CCI group, the CCI+DC group had significantly lower ICP, superior neurological and motor function at 24 h after injury, and less severe BBB damage after injury. Most inflammatory cytokine expressions and the number of apoptotic cells in the brain tissue of mice in the CCI+DC group were lower than in the CCI group at 3 days after injury, with markedly reduced astrocyte and microglia expression. However, the degree of brain edema in the CCI+DC group was greater than in the CCI group, and neurological and motor functions, as well as spatial cognitive and learning ability, were significantly poorer at 14 days after injury.

## Introduction

Traumatic brain injury (TBI) is an important cause of morbidity and mortality worldwide (1–3). It accounts for an estimated 37% of all injury-related deaths in the EU and 30.5% in the USA, and the mortality is estimated to be ~13 cases per 100,000 people in China (4, 5). The complex pathophysiological changes that occur after TBI, which result from primary and secondary injuries, can lead to increased intracranial pressure (ICP), brain contusion, brain edema, and blood–brain barrier (BBB) disruption (6–8). More importantly, TBI can induce temporary or permanent motor, sensory, cognitive, and emotional impairments that lead to poor prognosis (9, 10).

Decompressive craniectomy (DC) is an important treatment for patients with uncontrollable brain swelling, and is widely accepted for ICP reduction (11–14). Although DC is currently recognized as an effective means of reducing intracranial hypertension, whether it is beneficial for the treatment of moderate and severe TBI is still controversial, and there is no consensus on DC after moderate and severe TBI (15). Proponents believe that removal of a bone flap and the contused brain tissue can quickly relieve intracranial hypertension and reduce inflammation. However, those who prefer conservative treatment believe that, except in some emergency situations, surgery itself is traumatic to patients, excision of contused tissue will itself destroy surrounding brain tissues and aggravate neurological deficits, and overly aggressive surgical treatment is not conducive to good long-term prognosis.

Due to the paucity of clinical data, evaluations of DC after severe TBI are mostly retrospective. Moderate and severe TBI are emergencies; the injury mechanism, location, and degree of the injury vary greatly, and the surgical techniques of chief surgeons differ. All these factors substantially limit the evaluation of the effect of DC after TBI. Compared with clinical data, animal model evaluation has more consistency and broader evaluation indicators. However, despite its relevance, this issue has rarely been evaluated in experimental animals (16, 17).

**Figure 1:**
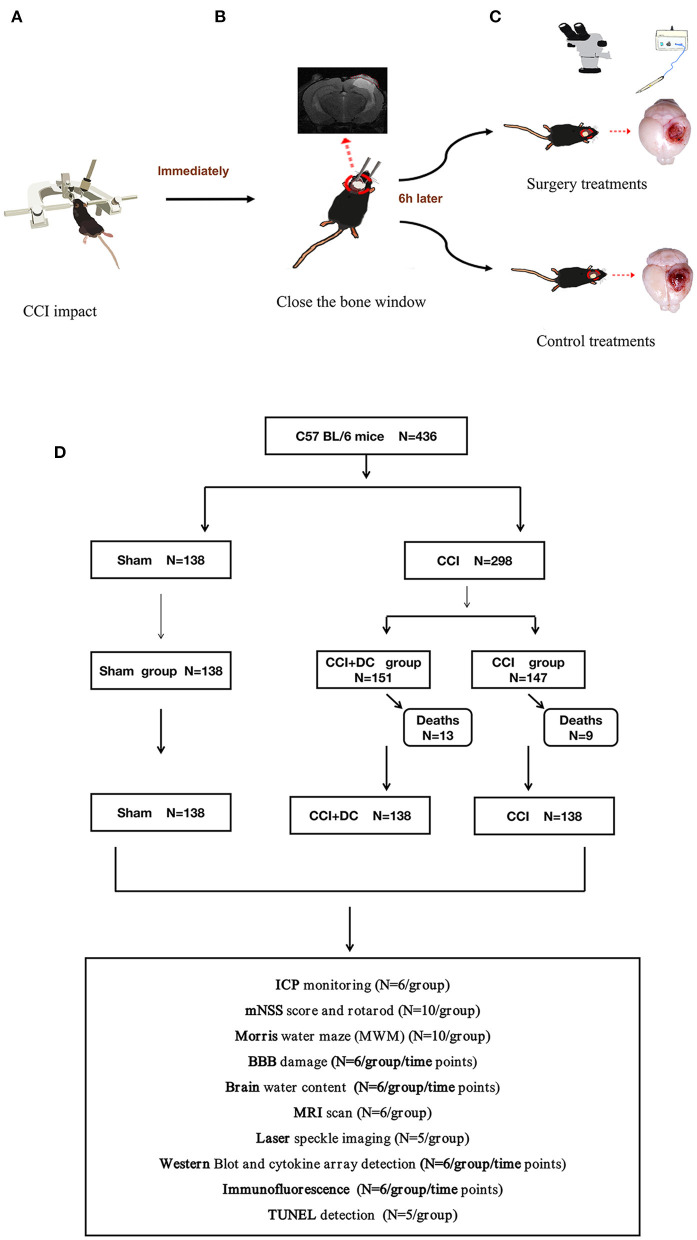
Experimental flow chart. **(A)** Mice in the CCI+DC group and CCI group were hit by CCI. **(B)** After the attack, the bone window was closed immediately. **(C)** 6 h after the attack, the mice in the CCI+DC group underwent bone window removal and contused tissue removal, the CCI group received the same anesthesia and skin incision. **(D)** Flow chart showing the experimental protocol with the number of animals used, died and included in the study.

This study established a stable animal model of DC in mice and compared the CCI+DC mice with the conservative CCI mice with regard to outcomes such as ICP, neurological function, inflammation, brain edema, and BBB function, providing references for clinical decision-making (Figure 1).

## Methods and materials

### Animals

Male C57BL/6 mice (aged 7–9 weeks, 22–24 g, Vital River Laboratory Animal Technology Co., Ltd., Beijing, China) were housed in the animal facility of Tianjin Medical University General Hospital under a 12-h light/dark cycle in a temperature-controlled room (20 ± 2°C) and were provided food and water *ad libitum*. All experimental procedures involving animals were approved by the Animal Ethics Committee of Tianjin Medical University (Tianjin, China).

### TBI model

**Figure 2:**
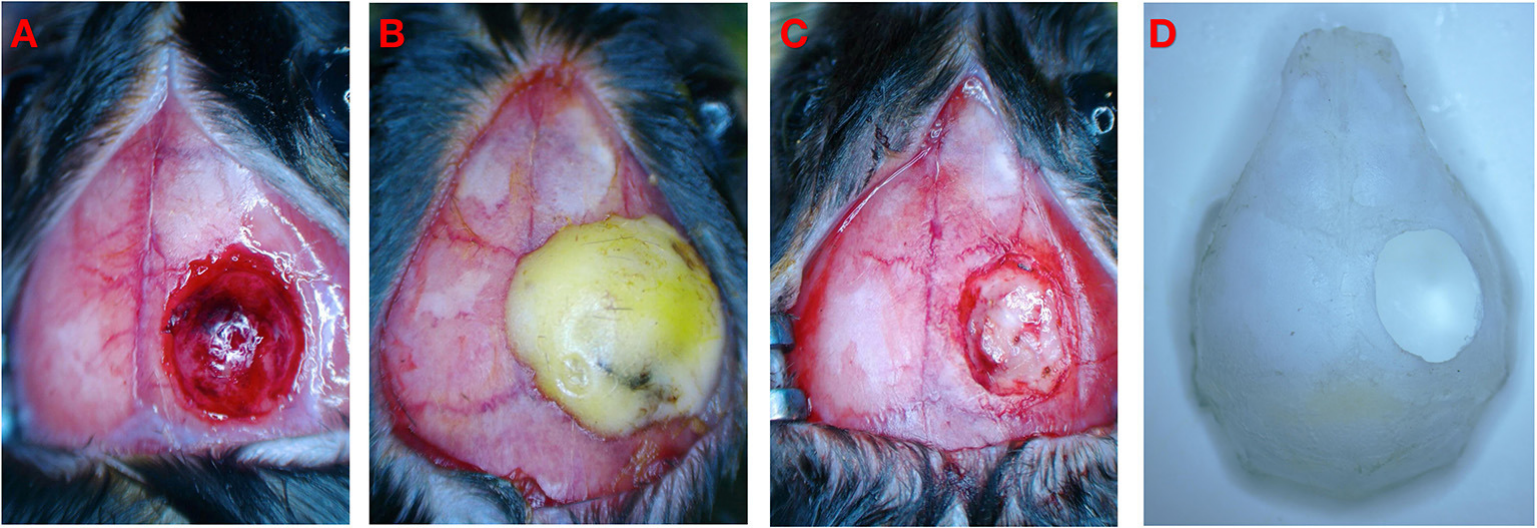
Schematic diagram of experimental operation. **(A)** Brain injury after CCI. **(B)** Close bone window after CCI. **(C)** CCI+DC group underwent bone window removal and contused tissue removal. **(D)** Schematic diagram of bone window.

The TBI mouse model was induced using a controlled cortical impact (CCI) device (eCCI-6.3 device, Custom Design & Fabrication, USA) as follows (Figure 2A). The mouse was anesthetized with a mixture of ketamine (100 mg/kg) and xylazine (10 mg/kg) by an intraperitoneal injection. After the head was shaved and disinfected, a 10-mm median incision was made on the scalp, and the periosteum was removed. The mouse was held on a fixed frame, and a 4 mm round bone window was cut in the center of the parietal bone (Figure 2D). During the process, we aimed to maintain bone flap integrity and avoid damage to the dura mater and brain parenchyma. We adjusted the position of the impactor tip to fit only the exposed dura mater for a single impact with these specific parameters (18), namely, flat impactor tip diameter: 3.0 mm, depth: 2.0 mm, speed: 5 m/s, and dwell time: 200 ms.

The mouse was then quickly removed from the fixator. As previously described by Zweckberger et al., we closed the bone window immediately after CCI to simulate the clinical response of elevated ICP under a nearly closed bone window after trauma (19, 20). If the bone flap is not replaced, the open craniotomy defect will provide a release mechanism for the contused-swollen brain preventing the reproduction of a precise model (20). In our study, the complete bone flap was immediately placed back into the bone window before the lesion was completely swollen and edematous. The bone window was fixed with Type I glass ionomer cement (Shanghai Medical Instruments Co., Ltd., China) to completely cover the bone window and the surrounding 1 mm area (Figure 2B). After the cement was completely dried and solidified, the surgical field was disinfected, and the scalp incision was closed with 4-0 sutures. The animal was placed in a thermostatic cage to maintain body temperature for 45 min, and then placed in an ordinary cage for separate feeding. All mice were carefully observed for ≥4 h after surgery and then daily.

### Experimental grouping and DC

Each mouse was randomly allocated to one of the three groups, namely, CCI+DC group, CCI group, and Sham group. Of these, the CCI+DC and CCI groups received CCI. The Sham group did not receive CCI; however, the other operations, including anesthesia, incision, bone window opening, and closure, were the same as in the CCI group. DC was performed in the CCI+DC group 6 h after CCI. After the mice were re-anesthetized and disinfected, the scalp and bone window were opened and the wound focus and surrounding area were disinfected. Under the microscope, the dura mater was broken by micro-tweezers to expose the swollen brain tissue, and small tampons prepared in advance were used to gently staunch. Lesions with obvious contusion and necrosis were removed using unipolar electrocoagulation (Yangzhou Xiaguang Medical Instrument Co., Ltd China). During such an intervention, movements should be slow and careful, and attention should be paid to hemostasis. For a small amount of slow bleeding, monopolar electrocoagulation should be performed while avoiding contact with the normal brain tissue. Hemostatic yarn (SURGICEL, Absorbable Hemostat Johnson & Johnson, USA) was used to stop bleeding (Figure 2C). After disinfection, the scalp incision was sutured. Animals were placed in a thermostatic cage to maintain the body temperature for 45 min, and were then placed separately in a cage. The mice in the CCI group underwent the same anesthesia process; however, no further treatment of the bone window and trauma was performed.

### ICP monitoring

**Figure 3:**
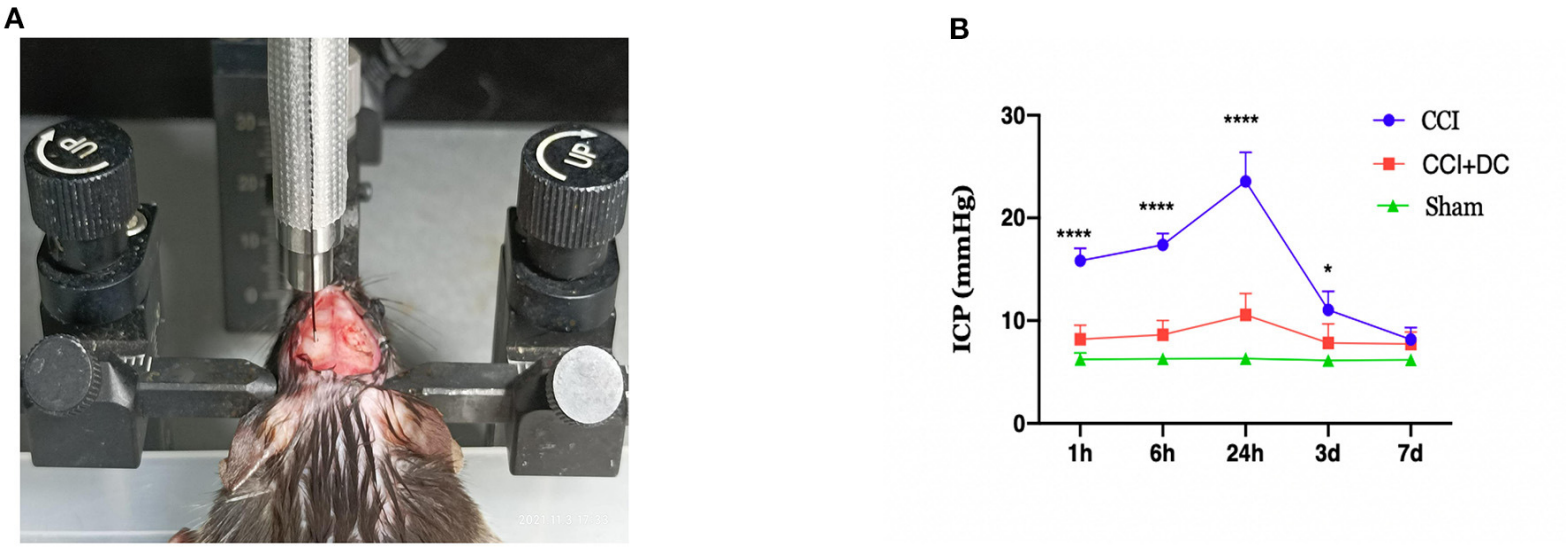
Intracranial pressure monitoring in mice. **(A)** Diagram for monitoring ICP in mouse. **(B)** Time-varying curves of ICP of mice in each group. Compared with the sham group, the ICP of the CCI+DC group and the CCI group was higher than that of the sham group after CCI, and the ICP of the CCI group was significantly higher than that of the CCI+DC group at 1, 6, and 24 h after attack (*n* = 6/group, *P < 0.05, ****P < 0.0001 vs. CCI group).

Intracranial pressure was monitored at 1 h, 6 h, 24 h, 3 days, and 7 days after CCI using the Transonic Scisense SP200 Data Acquisition System (Transonic, USA). The mice were anesthetized and fixed on the stereotactic frame, and a bone aperture of 0.8 mm diameter was created at 2.5 mm to the right of midline and 2.5 mm anterior to lambda. After calibration of the ICP monitoring device, a 1.6F piezoelectric flexible pressure probe (Transonic FTH-1211B-0012) was inserted 2 mm below the cerebral cortex through the bone aperture (Figure 3A). Real-time ICP data were observed using LabScribe (iWorx Systems, Inc., Dover, NH, USA). ICP was recorded at a sampling speed of 1.0 sample/s. The data were recorded and saved, and the average value within the observation period was used as the recorded value. The burr hole for ICP monitoring was sealed with Vetbond Tissue Adhesive (3M Corp., Maplewood, MN, USA) before skin closure. For ICP measurements at 6 h, 24 h, 3 days, and 7 days after injury, the same burr hole was accessed again using the same 0.8 mm drill bit (16).

### Modified neurological severity score and rotarod performance test

The Modified Neurological Severity Score (mNSS) and rotarod performance were evaluated as previously reported (21). The mNSS is a composite of the motor (muscle status and abnormal movement), sensory (visual, tactile, and proprioceptive sensibilities), beam balance, reflex absence, and abnormal movement tests, with a scoring standard between 0 and 18 points (normal score is 0 points; maximum score is 18 points). The higher is the score, the worse is the sensorimotor function. Mice were scored for mNSS on days 1, 3, 7, and 14 after CCI by the staff unaware of the experimental grouping.

Mouse exercise capacity was evaluated using rotarod, which is a rotating bar fatigue tester (YLS-4C; Yima Optoelec Co., Ltd., Beijing, China), at the same time as mNSS scoring. Mice were given 3 min for acclimation to the instrument rotating at 10 rpm. Acceleration from 0 to 40 rpm was then initiated for 5 min, and the fall time (or the time to complete five effortless spins) was recorded for each mouse. If mice could run in a normal posture for >5 min, the fall time was calculated as 300 s. A total of three tests were performed at each time point, with a 30-min interval, so that the mice could adequately rest. The average of the three trials was taken as the data of that time point.

### Morris water maze

The Morris water maze (MWM) was applied 14 days after CCI. In brief, the mice in each group were randomly numbered and trained to locate the hidden platform for 6 days. They were trained four times a day with different starting positions, each time with an interval of ≥30 min, and the starting positions randomly changed daily. Whether the mice found the platform within 90 s or not, they were allowed to stay on the platform for 15 s to memorize the surrounding environment and the location of the markers on the wall. On day 7, the platform was removed and the mice were placed randomly for 90 s of free exploration. The operator of the experiment ensured that the specific mouse grouping was unknown to reduce bias. The latency to reach the platform and the target quadrant, the time spent in the target quadrant, and the number of times the platform was crossed were automatically determined by the software. After each experiment, the mice were dried with absorbent towels and placed in a constant-temperature cage with dry bedding to assure warmth.

### Blood–brain barrier damage

Blood–brain barrier integrity was measured using Evans blue (EB) extravasation according to a previous study (22). The mice were anesthetized with 7% chloral hydrate and injected into the tail vein with 100 μl of 2% EB (E2129; Sigma-Aldrich, St. Louis, MO, USA) on days 1, 3, 7, and 14 after CCI. Two hours after injection, the mice were euthanized and immediately perfused with cold phosphate-buffered saline (PBS). Brain tissue samples from the injured side were cut into pieces in a tube. After adding 1 ml of formamide to each tube, samples were extracted in a 60°C water bath for 24 h. The tubes were then centrifuged at room temperature at a speed of 4,000 *g* for 15 min, and 200 μl of the supernatant was added to a 96-well plate. The scale was constructed by diluting EB with formamide in a concentration range of 25.6–0.4 μg/ml. The OD value of each well was detected by absorbance spectroscopy at 610 nm. The EB concentration of each supernatant was calculated.

### Brain water content and MRI scan

The water content of brain tissue was detected as previously described (23, 24). In brief, mice were euthanized 1, 3, 7, and 14 days after CCI. Immediately thereafter, brain tissues were extracted, weighed, and recorded in a small dish as the wet weight, and then dried at 70°C for 48 h in the oven, weighed, and recorded as the dry weight. The degree of brain edema was evaluated as (wet weight – dry weight)/wet weight. The result is shown as a percentage.

T2-weighted imaging was performed on days 1, 3, 7, and 14 after CCI with a 9.4 T high-field MRI scanner (BioSpec 94/30 USR; Bruker, Billerica, MA, USA). The scanning parameters included 800 μm slice thickness. After MR scanning, the area of edema and necrosis was measured according to the T2 images, and a matching region of interest (ROI) was manually created using the RadiAnt DICOM Viewer (Medixant, Poznan, Poland). The total contusion volume and edema area are expressed as the sum of the ROI areas multiplied by the thickness in each scanning plane (25, 26).

### Laser speckle imaging

On days 1, 3, 7, and 14 after CCI, the cortical blood flow of mice in each group was evaluated using the PeriCam PSI System (Perimed AB, Sweden). First, the mouse skull was fully exposed under anesthesia. The cortical blood flow of the traumatic and contralateral sides was evaluated using a laser speckle imager (detection distance: 10 cm, laser irradiation area: 2 × 2 cm, 1,386 × 1,034 pixels, regional spatial contrast calculated by 3 × 3 secondary matrix), in a process lasting for 2 min. After detection, the skin was sutured and disinfected. PIMsoft 1.2 was used to analyze perfusion data. Each detection result selects the same ROI position and size and analyzes the average measured values at the same time.

### Western blot and cytokine array detection

On days 1, 3, 7, and 14 after CCI, brain tissue proteins were extracted, and cytokines were detected by western blot and array. In brief, immediately after euthanasia, mice were perfused with cold PBS, and then, the injured lateral brain tissue was obtained. The total protein was extracted with RIPA protein lysis buffer and 1% PMSF. The protein sample was separated by 10% SDS-PAGE at 120 V for 90 min, and the separated proteins were transferred onto PVDF membranes. The PVDF membranes were then blocked, cut into bands, and incubated with primary antibodies {β-actin Proteintech 66009, 1:5,000; IL-1-β [CST (3A6) Mouse mAb #12242], 1:250; IL-6 (antibody ab9324), 1:1,000} overnight at 4°C on a shaker. After washing three times, the bands were incubated with an HRP-coupled secondary antibody (1:5,000, Zhongshanjinqiao China) for 1 h at room temperature. A ChemiDoc imaging system (Bio-Rad, Hercules, CA, USA) was used to detect the exposure by ECL chemiluminescence.

Cytokine array was performed as per manufacturer's instructions (ARY006; R&D Systems, Minneapolis, MN, USA). The brain tissue proteins of the abovementioned groups were mixed according to the proportion of total protein content (six mixed with one), and each protein detection chip membrane was incubated overnight at 4°C with 2,000 μg of the total protein content. First, after sufficient washing with 1 × washing buffer, each chip protein was incubated with streptomycin-horseradish peroxidase for 30 min. After a new wash, each chip was immersed in ECL luminescent solution for 1 min and then exposed and detected by the ChemiDoc imaging system (Bio-Rad).

### Immunofluorescence and TUNEL detection

After euthanasia and heart perfusion with cold PBS and 4% PFA, the intact brain tissue was obtained and fixed in 4% PFA at 4°C for 24 h. Next, the brain tissue was dehydrated in 15% and 30% sucrose, embedded with OCT, and cut into coronal sections with an 8 μm thickness using a microtome for sectioning frozen sections (CM 1950; Leica Biosystems, Deer Park, IL, USA). After rewarming, the OCT was washed away with PBS and the tissue was circled with a hydrophobic pen. Then, the brain sections were incubated with 2% BSA, 2% goat serum, 0.2% Triton X-100, and 0.05% Tween 20 in PBS for 1.5 h at room temperature. After removing the liquid, 30 μl of diluted primary antibody [anti-Neu N ab177487 (1:300), anti-GFAP ab4674 (1:1,000), and anti-Iba 1 ab5076 (1:200)] were added to each tissue and incubated overnight at 4°C. After rewarming and washing with PBS, the fluorescent-labeled secondary antibody was added and incubated at room temperature for 1 h, while avoiding exposure to light. Then, the sections were sealed with DAPI-containing sealing tablets. Once ready, the samples were observed with an inverted fluorescence microscope (Olympus, Shinjuku City, Tokyo, Japan).

After the incubation with Neu N primary antibody and fluorescent secondary antibody, the TDT enzyme from the TUNEL staining kit (Roche, 11684817910) was mixed with fluorescent labeling solution in a ratio of 1:9. After 10 min, 30 μl of the mixed solution was dripped onto each tissue, which was then incubated at 37°C for 1 h, washed with PBS, sealed with DAPI-containing sealing tablets, and imaged with an inverted fluorescence microscope (OLYMPUS). ImageJ software was used to calculate the gray values used for measuring staining intensity and nuclei in the observation area.

### Data analysis

All data are expressed as mean ± standard error of the mean. Independent unpaired *t*-tests and two-way ANOVA were used to evaluate significance, and *p* < 0.05 was considered statistically significant. SPSS 22.0 software (IBM Corp., Armonk, NY, USA) and GraphPad Prism 9.0 (GraphPad Software, San Diego, CA, USA) were used for data analysis and plotting.

## Results

### DC can effectively reduce ICP in severe TBI

The ICP was dynamically monitored at 1 h, 6 h, 24 h, 3 days, and 7 days after CCI. Compared with that of the Sham group, ICP in the CCI group increased gradually over time, reached a peak at 24 h after CCI, and then slowly decreased. Compared with that of the CCI group, ICP in the CCI+DC group was significantly lower at each observation time point, and there were significant differences at 1 h, 6 h, 24 h, and 3 days (*p* < 0.05) (Figure 3B).

### DC can improve neurological and motor function at 24 h after CCI, but weaken the spatial cognition and learning ability at late stages of CCI

**Figure 4:**
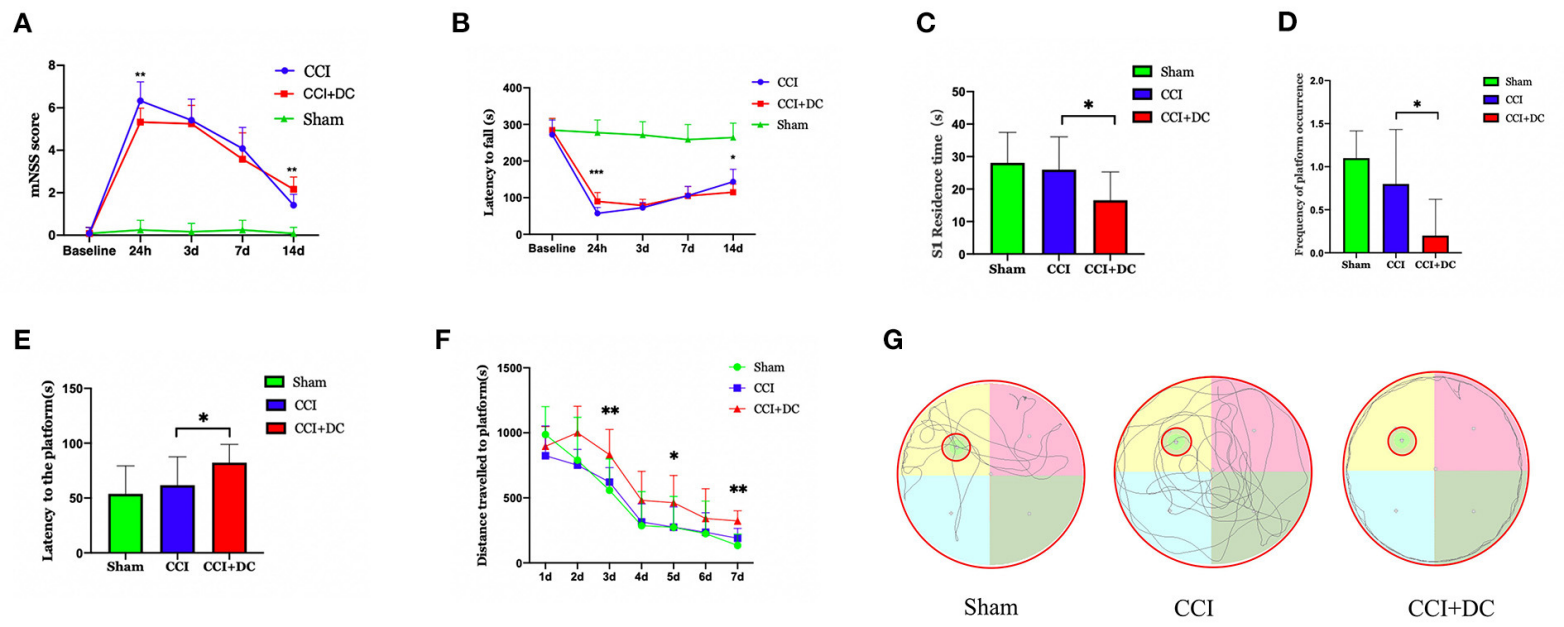
Functional results. **(A)** Comparison of results among all groups indicated that mNSS score of mice in the CCI+DC group improved 24 h after CCI compared with that in the CCI group, while functional score of mice in the CCI+DC group decreased significantly as time went by 14 days after CCI compared with that in the CCI group, and the difference was statistically significant. **(B)** The results of the rotarod indicated that the latency to fall in the CCI+DC group was significantly improved compared with that in the CCI group at 24 h after CCI, and the difference was statistically significant. However, on the 14th day after CCI, the motor function of mice in the CCI+DC group was worse than that in the CCI group, and the difference was statistically significant. **(C)** MWM results indicated that on 7th day of the MWM experiment, the time for mice in the CCI+DC group to pass S1 quadrant. **(D)** The number of platforms were significantly lower than those in the CCI group, and the differences were statistically significant. **(E)** MWM results indicated that the platform latency of mice in the CCI+DC group was significantly higher than that in the CCI group, and the difference was statistically significant. **(F)** The total movement distance of mice was counted in the MWM experiment. It was found that the movement distance of mice in the CCI+DC group was longer than that of mice in the CCI group on day 3, 5, and 7 of the water maze experiment, and the difference was statistically significant. **(G)** Movement route of mice on day 7 between different group (*n* = 10/group, *P < 0.05, **P < 0.01, vs. CCI group).

At 24 h after CCI, the mNSS scores in the CCI+DC group were significantly lower than those in the CCI group (*p* < 0.05). However, there was no significant difference in mNSS scores between the CCI and CCI+DC groups at 3 and 7 days after CCI. By the 14th day after injury, compared with the CCI group, the mice in the CCI+DC group had significantly higher mNSS score (*p* < 0.05) (Figure 4A).

Similarly, in the rotarod test, compared with the CCI group, the CCI+DC group had a longer latency time before falling at 24 h after injury (*p* < 0.05). There was no significant difference in latency time between the two groups at 3 and 7 days after CCI. By the 14th day, the CCI group had a longer residence time (*p* < 0.05) (Figure 4B).

The MWM was used to evaluate spatial cognition and learning ability 14 days after CCI. After removing the hidden platform, compared with that of the CCI group, the platform latency period of the CCI+DC group was longer, and the residence time in the target quadrant and the number of times crossing the platform area were significantly lower (*p* < 0.05) (Figures 4C–E). Moreover, in the water maze experiment, on the 3rd, 5th, and 7th days, the movement distance in the CCI+DC group was longer than that of the CCI group, ((Figure 4G) and the total movement distances of the two groups were significantly statistically different (*p* < 0.05) (Figure 4F).

### DC can reduce BBB damage in severe TBI

**Figure 5:**
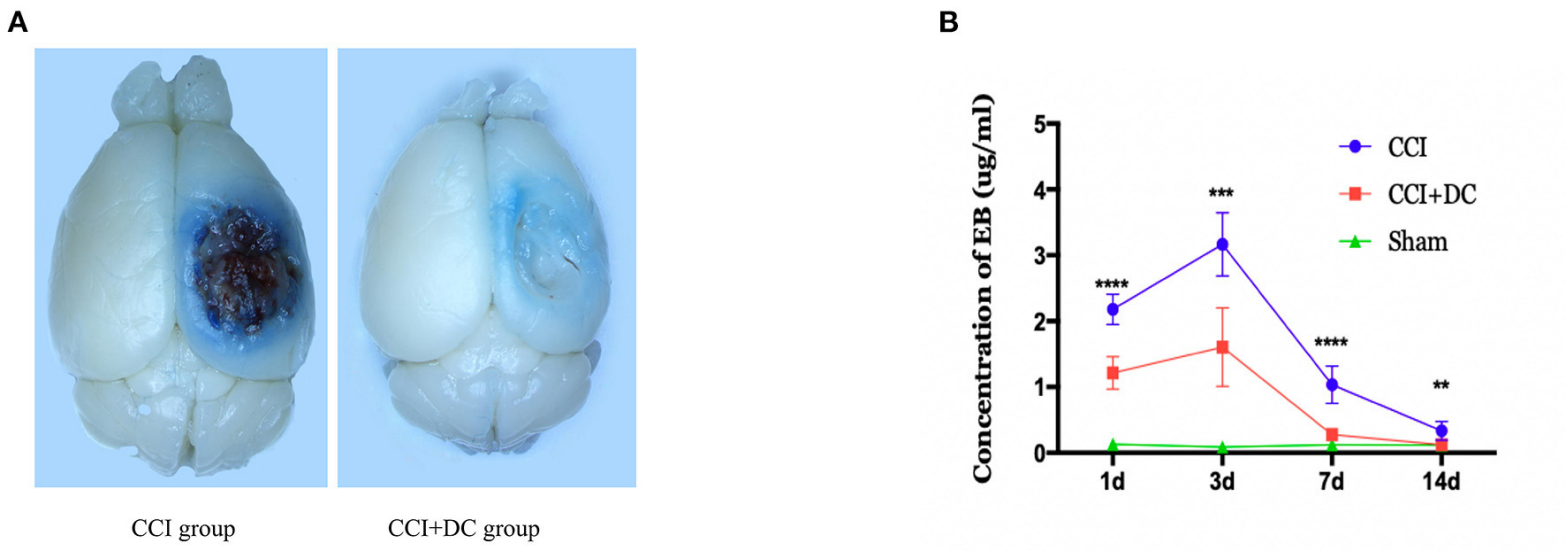
Effects on the blood-brain barrier. **(A)** General view of brain tissue after EB tail injection in rmice on 3 days after traumatic brain injury. **(B)** EB was extracted from the brain tissue to calculate the infiltration content of EB, and the results indicated that the infiltration amount of EB in the CCI+DC group was significantly reduced compared with the CCI group at each time point after CCI, with significant statistical difference (*n* = 6/group, **P < 0.01, ***P < 0.001, ****P < 0.0001 vs. CCI group).

The EB leakage test was used to evaluate BBB damage in the CCI+DC and CCI groups at different time points after CCI (Figure 5A). On the 3rd day after CCI, the leakage of EB into the brain tissue of the CCI+DC and CCI groups reached a peak and gradually decreased over time. Compared with that of the CCI group, EB leakage in the CCI+DC group was significantly lower at 24 h, 3 days, 7 days, and 14 days after CCI (*p* < 0.05) (Figure 5B).

### DC can reduce brain tissue edema and necrosis but can briefly aggravate whole-brain edema in severe TBI

**Figure 6:**
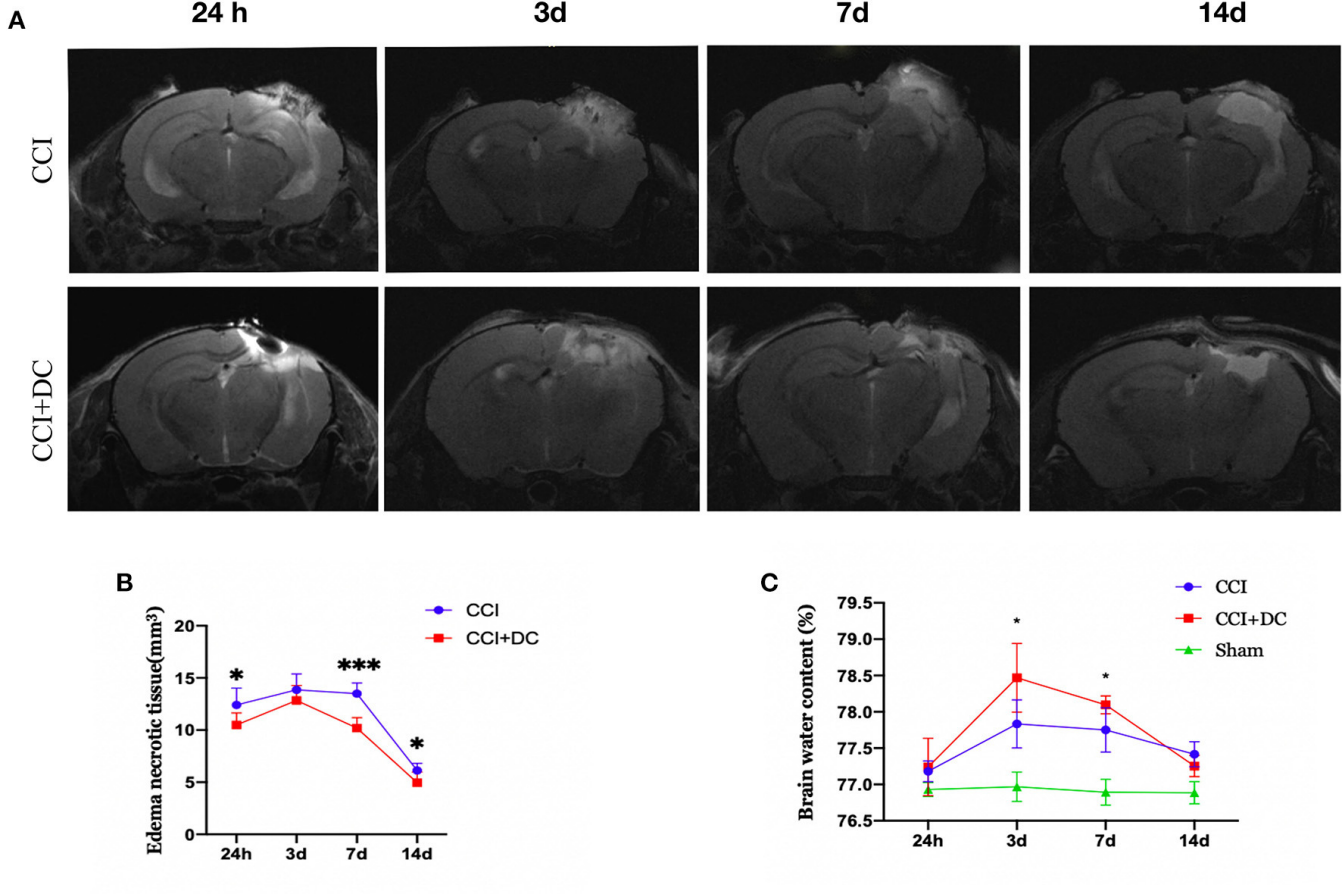
Effect on cerebral edema. **(A)** MR examination was performed at different time points after CCI. **(B)** The statistical results were shown intracranial edema and contusion tissues of mice in the CCI+DC group at each time point after CCI was lower than that of the CCI group, and the difference was statistically significant on the first, 7th and 14th day after CCI. **(C)** Brain water content was detected by extracting brain tissue, as shown in the **(C)** on the 3rd and 7th day after CCI, the degree of brain edema in the CCI+DC group was significantly worse than that in the CCI group, and the difference was statistically significant (*n* = 6/group, *P < 0.05, ***P < 0.001 vs. CCI group).

The brain water content, measured to evaluate brain edema, showed obvious edema in the CCI and CCI+DC groups at 24 h after CCI, reaching its peak on the 3rd day, and then gradually subsiding, leaving only a liquefied focus on the 14th day after CCI. On the 3rd and 7th days after injury, the brain water content in the CCI+DC group was significantly higher than that in the CCI group (*p* < 0.05) (Figure 6C).

However, MRI results showed that the edema and necrotic zone of mice in the CCI+DC group were significantly smaller in size than that in the CCI group. Especially on the 1st, 3rd, and 14th days after CCI, there was a significant statistical difference between the two groups (*p* < 0.05) (Figures 6A,B).

### DC can improve brain cortex perfusion in severe TBI

**Figure 7:**
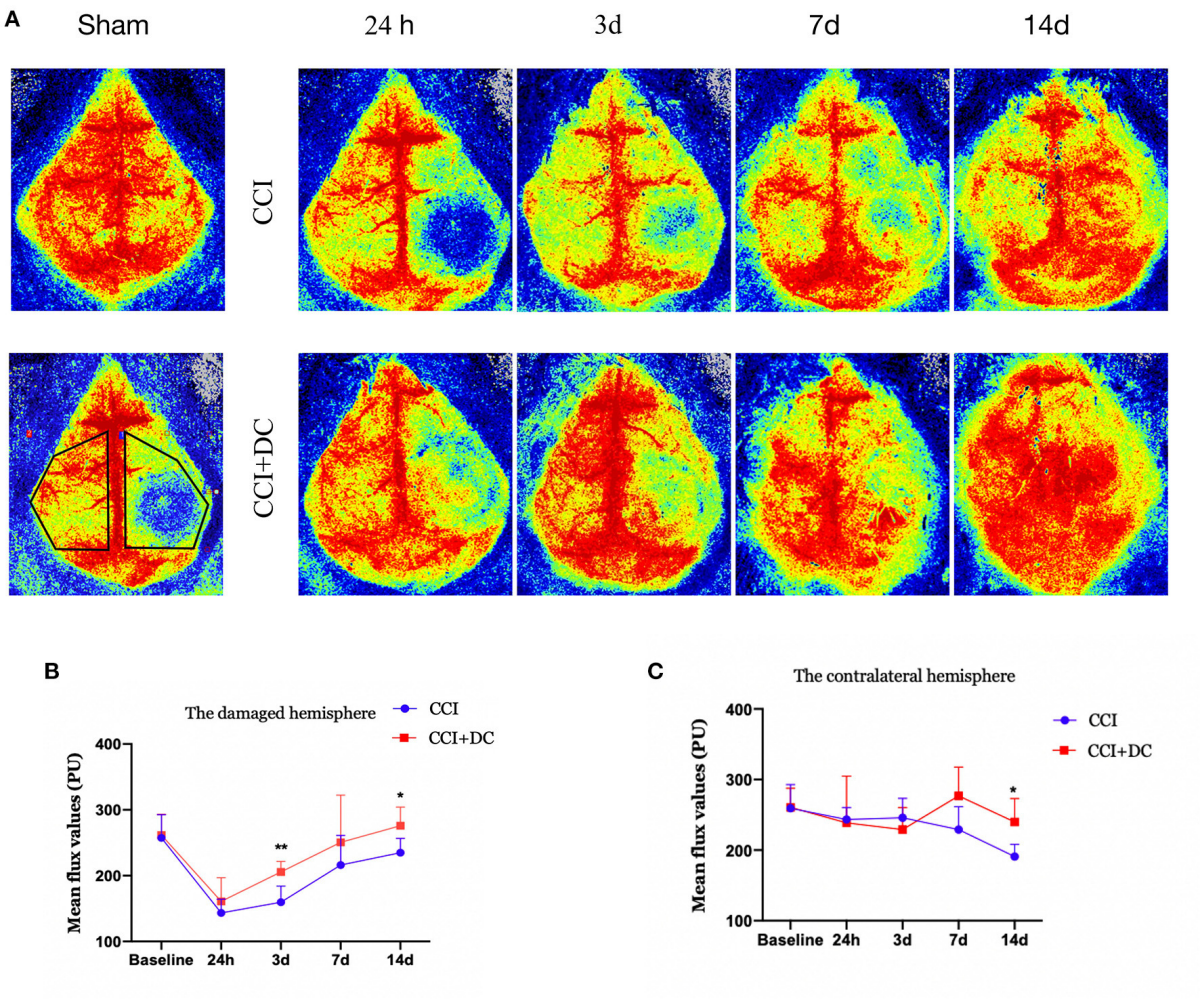
Effects on cerebral blood flow. **(A)** Laser speckle test was performed at different time points after CCI to evaluate cerebral blood flow, and the statistical results were shown in **(B,C)**. **(B)** It was statistically found that the cerebral blood flow on the injured side of the CCI+DC group after CCI was higher than that of the CCI group, and the results were statistically different on the 3rd and 14th day after CCI. **(C)** Statistically, cerebral blood flow in the healthy side of the CCI+DC group was higher than that in the CCI group on the 7th day after CCI, and the difference was statistically significant (*n* = 5/group, *P < 0.05, **P < 0.01, vs. CCI group).

Perfusion in the cortex was evaluated by laser speckle imaging. Blood flow in the CCI+DC and CCI groups gradually increased over time. Compared with that of the CCI group, perfusion of the injured cortex in the CCI+DC group was greater on the 1st, 3rd, 7th, and 14th days after CCI, especially on the 3rd and 14th days (*p* < 0.05) (Figures 7A,B). Similarly, for the contralateral cerebral hemisphere, cortical blood flow in the CCI+DC group was higher than that in the CCI group on the 7th and 14th days, especially on the 14th day (*p* < 0.05) (Figures 7A,C).

### DC can reduce inflammation, apoptosis, and glial cell expression after severe TBI

**Figure 8:**
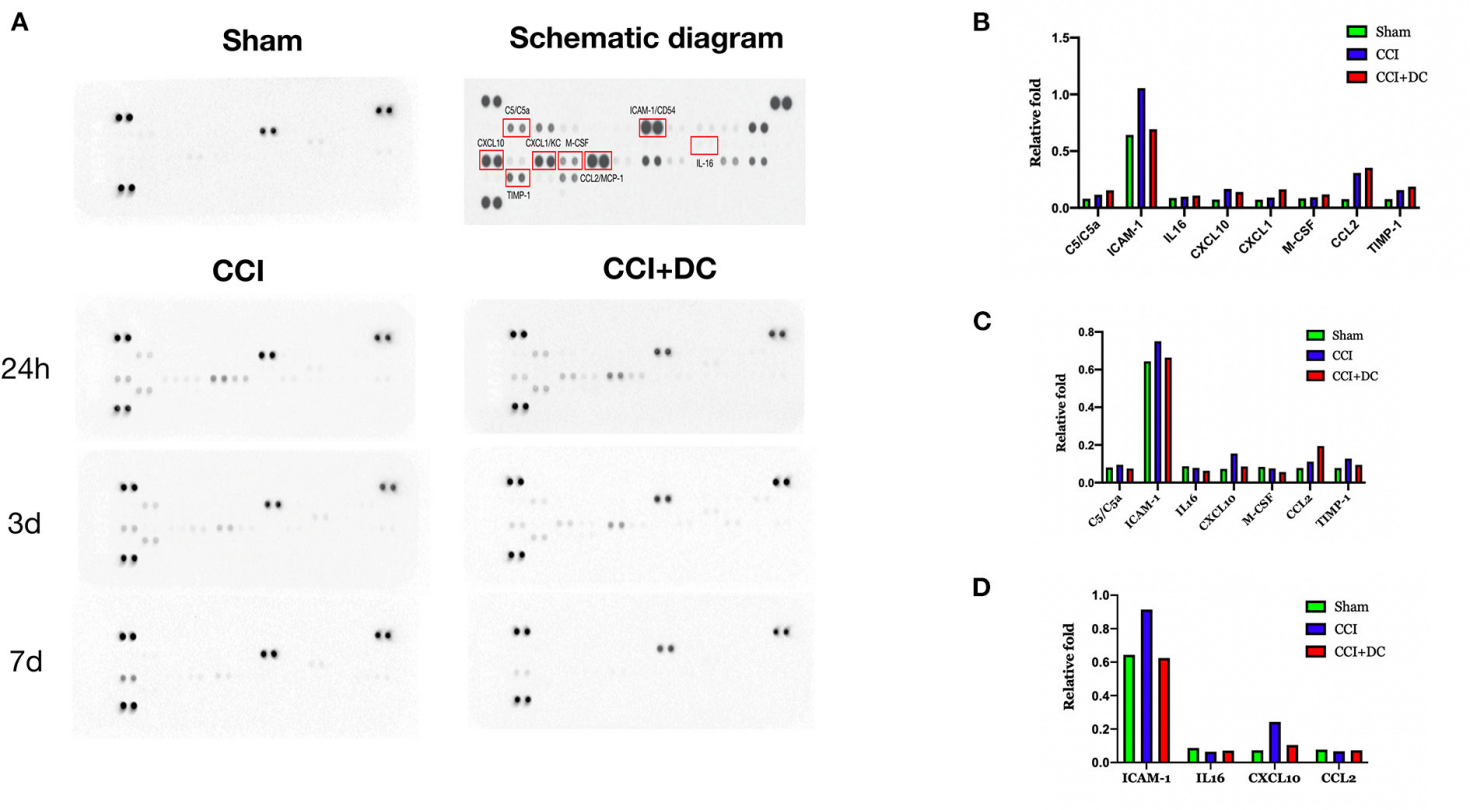
Changes in the expression of inflammatory factors. **(A)** Changes in the expression of inflammatory factors at 24 h, 3 d, and 7 d after brain trauma. **(B)** Expression levels of inflammatory factors in different groups of mice at 24 h after brain trauma. **(C)** Different groups of mice The expression level of inflammatory factors on the 3rd day after brain injury. **(D)** Expression levels of 7D inflammatory factors in mice in different groups of D after brain trauma.

A cytokine array was used to detect the levels of inflammatory factors in the brain tissue of mice in each group. Compared with that of the Sham group, the expression of inflammatory factors in the brain tissue of TBI mice reached its peak 24 h after TBI, and then gradually decreased. On the 1st day after CCI, the levels of inflammatory cytokines, namely, C5/C5a, IL-16, CXCL1 (KC), M-CSF, CCL2 (MCP-1), and TIMP-1 were higher in the CCI+DC group than in the CCI group, while the levels of ICAM-1 (CD54) and CXCL10 (IP-10/CRG-2) were lower (Figures 8A,B). However, the levels of C5/C5a, ICAM-1 (CD54), IL-16, CXCL10 (IP-10/CRG-2), M-CSF, and TIMP-1 in the brain tissue of the CCI+DC group were significantly lower than those in the CCI group on the 3rd day after CCI (Figure 8C), and ICAM-1 (CD54), IL-16, and CXCL10 (IP-10/CRG-2) levels were lower than those in the CCI group on the 7th day after CCI (Figure 8D).

**Figure 9:**
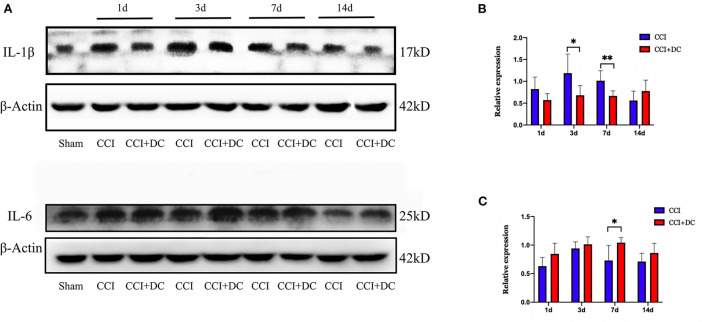
The effect on inflammatory factors. **(A)** Expression changes of inflammatory factors IL1β and IL6 on day 1, 3, 7, and 14 after CCI, and statistical results are shown in **(B,C)**. **(B)** ILIβ in the CCI+DC group was significantly higher than that in the CCI group on the 3rd and 7th day after CCI. **(C)** IL6 in the CCI group was lower than that in the CCI group on the 7th day after CCI, and the results were also statistically different (*n* = 6/group, *P < 0.05, **P < 0.01, vs. CCI group).

Meanwhile, western blotting was used to detect the expression levels of IL-1β and IL-6 in mouse brain tissue in each group. Compared with that of the CCI group, the expression level of IL-1β was significantly lower in the CCI+DC group, especially on the 3rd and 7th days (*p* < 0.05) (Figures 9A,B). However, the IL-6 expression level was significantly higher in the CCI+DC group than in the CCI group at maximum time points, especially on the 7th day (*p* < 0.05) (Figures 9A,C).

**Figure 10:**
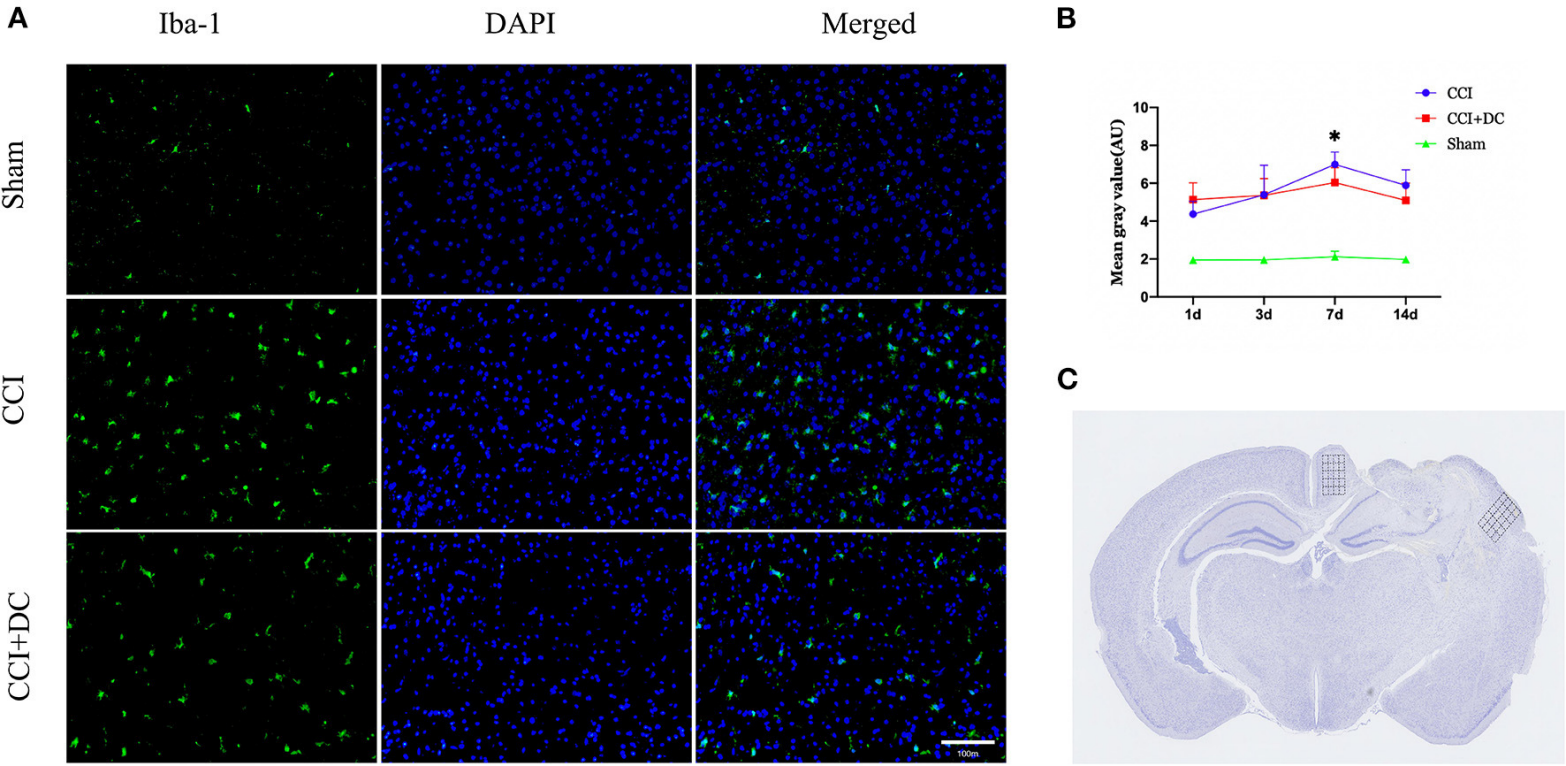
Effect on peri-contusional cortex microglia. **(A)** The expression of microglia in different groups on the 7th day after traumatic brain injury. **(B)** Expression of microglia in the CCI+DC group was significantly lower than that in the CCI group on the 7th day after CCI, the expression of cortical microglia was statistically different from that in the CCI group. **(C)** Detected areas of microglia and astrocytes. *n* = 6/group/time points, *P < 0.05, vs. CCI group.

**Figure 11:**
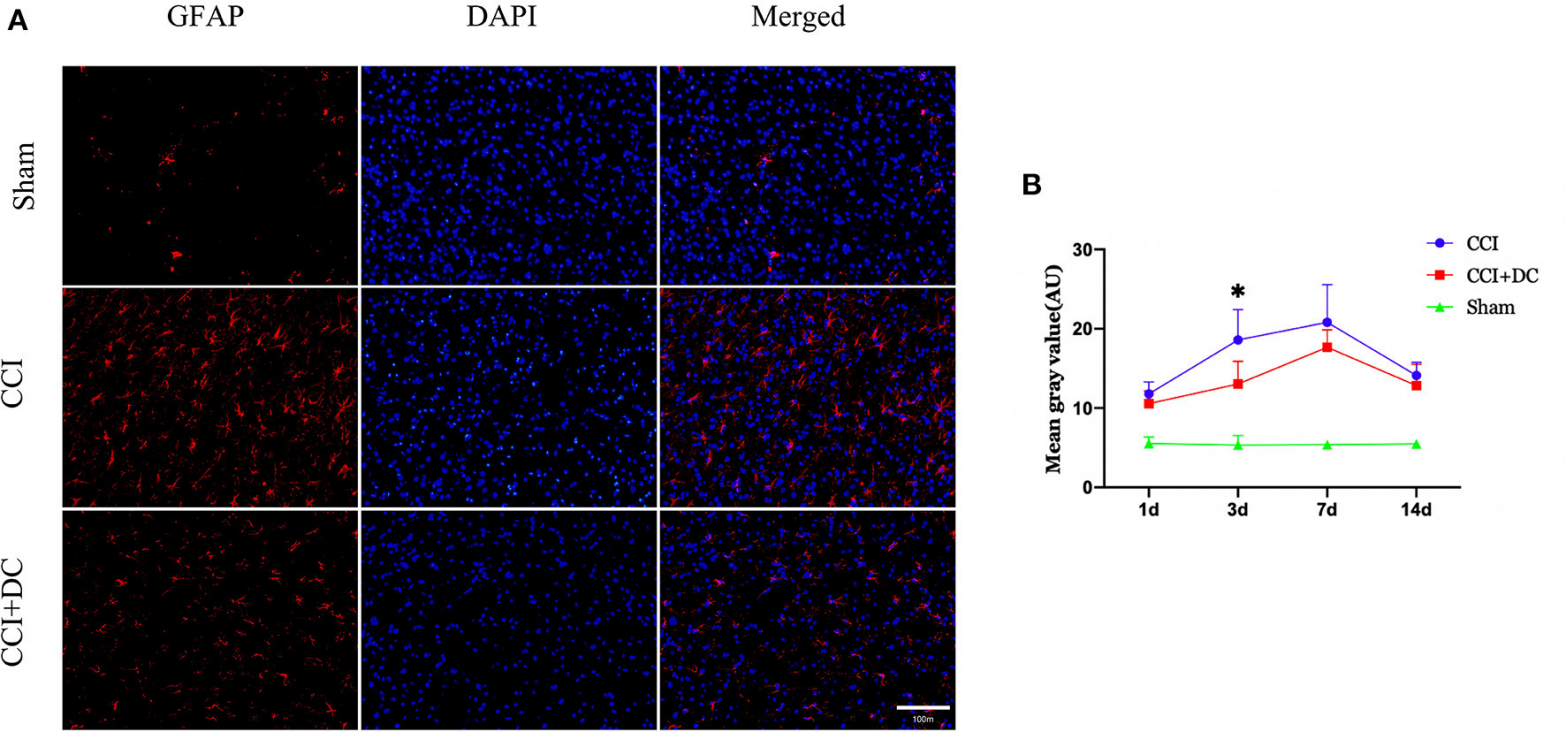
Effect on peri-contusional cortex astrocytes. **(A)** The expression of astrocytes in different groups on the 3rd day after brain injury. **(B)** On day 3 after CCI, the expression of microglia in the cortex of the CCI+DC group was significantly lower than that of the CCI group, and the expression of was statistically different from that in the CCI group (*n* = 6/group/time points, *P < 0.05, vs. CCI group).

Immunofluorescence was used to compare the expression levels of glial cells (Figure 10C). Compared with that in the CCI group, the number of microglial cells in the CCI+DC group was significantly reduced on the 7th day (*p* < 0.05) (Figures 10A,B). Moreover, compared with that of the CCI group, the expression of astrocytes in the CCI+DC group was significantly reduced, and the difference was statistically significant on the 3rd day (*p* < 0.05) (Figures 11A,B).

**Figure 12:**
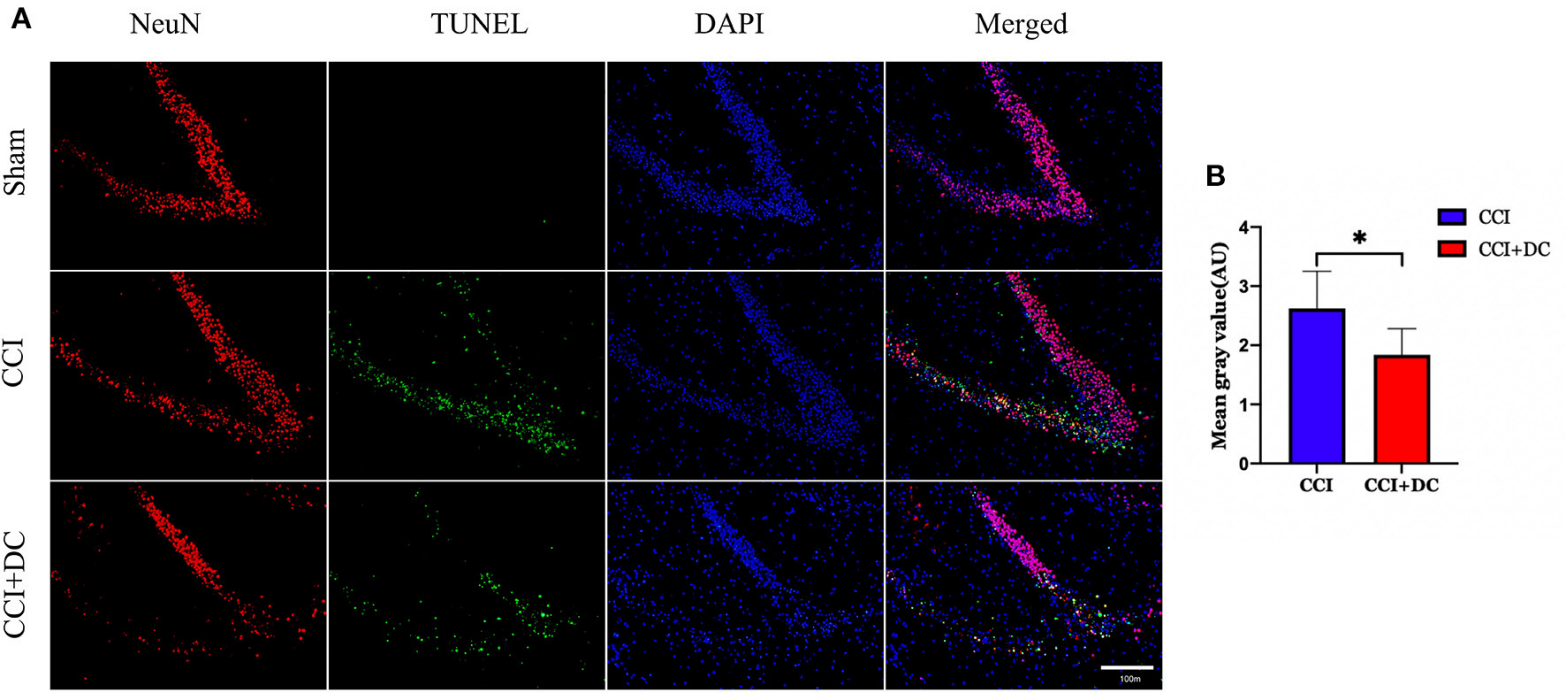
The effect of neuronal apoptosis in hippocampus on the third day after brain injury. **(A)** On the 3rd day after brain injury, the apoptosis of hippocampal neurons in different groups. **(B)** Hippocampal neurons apoptosis on the 3rd day after CCI. The results showed that the neuronal apoptosis of the CCI+DC group was less than that of the CCI group, and the difference was statistically significant (*n* = 5/group, *P < 0.05, vs. CCI group).

Furthermore, TUNEL staining was performed on nerve cells in the ipsilateral hippocampus on the 3rd day after CCI. The results showed significantly lower neuron apoptosis near the lesion in the CCI+DC group than in the CCI group (*p* < 0.05) (Figures 12A,B).

## Discussion

Decompressive craniectomy is widely used in clinical practice and plays a vital role in saving patients with life-threatening elevated ICP, and the use of DC will become increasingly common for severe head injuries and for other diseases such as stroke, subarachnoid hemorrhage, and infection. Thus, it is of great significance to establish a DC model and evaluate it systematically.

Although closed TBI models appear simpler, the depth and extent of the damage are not very reproducible. And most of the brain trauma caused by closed TBI is mild to moderate (17), in which case conservative treatments are usually used in the clinical process. At present, mouse models for weight drop injury (WDI), fluid percussion injury (FPI), and CCI have been created for studying severe brain trauma. Each model has several advantages and disadvantages. In WDI, the presence of the skull causes high variability in brain tissue changes. In addition, the mechanism of tissue damage associated with FPI is not similar to that observed in clinical TBI (27). The CCI model is an effective model to study TBI (28); this model uses an impactor resulting in significant neurological deficits as well as histopathological changes, including extensive cortical damage, hippocampal neuronal cell loss, extensive reactive gliosis, and breakdown of the BBB (18). And through closing the bone window after CCI, it can simulate the clinical situations of contused swollen tissues under a nearly closed bone window after trauma. Therefore, the CCI model is more consistent with the mechanism of clinical brain injury and can produce reproducible brain injury. Therefore, in our study, we chose to use the CCI mouse model to simulate patients with severe TBI who would need DC surgery in clinical practice. In our DC process, we chose a 4-mm bone window; this size can avoid the uncontrolled expansion of the brain caused by an excessively large bone flap, which may cause adverse neurological outcomes (29). In addition, compared with previous studies (16, 17), we cleared the apoptotic tissues to further release pressure in the intracranial space. By monitoring ICP at different time points after the TBI onset, we showed that the DC with focal debridement model had a clear decompressive effect in mice with TBI, as compared with the control group.

Studies have shown different results for the effects of DC on neurological recovery after TBI. Early clinical studies showed that DC has a positive effect on the recovery of neurological function after TBI (30–32). However, other studies showed that DC exacerbates functional impairment (17). Currently, two important multicenter, clinical RCT trials of DC have been conducted. The randomized early decompressive craniectomy (DECRA) trial that included 155 adults with TBI showed that early bifrontotemporoparietal DC decreased ICP and the length of stay in the ICU but was associated with more unfavorable outcomes (29), and 12 months after severe diffuse TBI, DC did not improve outcomes and increased the proportion of vegetative survivors (33). The Randomized Evaluation of Surgery with Craniectomy for Uncontrollable Elevation of Intracranial Pressure (RESCUEicp) trial with 408 TBI patients showed that, at 6 months, DC resulted in a lower mortality rate, a higher proportion of vegetative outcomes, lower severe disability, and upper severe disability than medical care. The rates of moderate disability and good recovery were similar between the two groups (34). Possible explanations for the different results for neurological recovery include different injury locations, different mechanisms, focal vs. diffuse injuries, different surgical indications in different experiments, and different surgeons for the DC operations. The damage caused to the brain tissue during the DC process itself is also a factor that should be considered.

Studies have shown that the BBB is disrupted after TBI (8, 35). BBB disruption is an early event that occurs within hours following injury but can persist for years (36). The destruction of the BBB can aggravate cerebral edema and increase ICP. After TBI, the increase in paracellular transport, indicated by a loss of tight junction proteins and an increase in transcytosis across the endothelium, contributes to BBB dysfunction; this leads to an influx of immune cells, such as neutrophils, and transport of large molecules and serum proteins that can exacerbate the inflammatory response (37, 38). In addition, intracranial astrocytes, endotheliocyte, and inflammatory factors play important roles in the destruction of the BBB (8, 39–41).

The early inflammatory response plays an important role in clearing tissue debris and repairing wounds. However, excessive inflammation can adversely affect neuronal function (42). For example, excessive inflammatory mediators such as IL-1β, IL-6, IL-18, and TNFα may lead to secondary injury after TBI (43). Although IL-1β alone does not seem to directly cause neuronal death in cell culture, in some cases, IL-1β can induce neuronal death, especially when other pro-inflammatory cytokines such as TNFα are present (44). Unlike the undesirable effects of IL-1β and TNFα in humans and animals, IL-6 appears to have neuroprotective effects in animal models (45).

Early studies have shown that after brain injury, cerebrovascular autoregulation is impaired or non-functional in many patients (46), and the brain blood volume decreases sharply after brain trauma (47). When cerebral edema occurs and ICP increases after TBI, the cerebral perfusion pressure will decrease, which leads to cerebral ischemia and hypoxia (48); DC can improve not only the ICP itself, but also cerebral blood flow and brain metabolism, as demonstrated in neuroimaging and multimodal studies (49, 50).

Early restoration of BBB integrity may aid in preventing the sequelae of other comorbidities associated with TBI, including post-traumatic epilepsy and neurodegenerative disease (51). During the DC process, the reduction of ICP and the clearance of contused necrotic tissue reduce oxidative stress and inflammation, thereby reducing post-TBI BBB damage and improving cerebral blood flow. However, during DC, the removal of severely contused and necrotic tissue aggravates the neurological injury. This change is associated with secondary injury caused by DC surgery.

Astrocytes and microglia have a dual effect on the recovery of brain tissue (52–56). Reactive astrocytes are capable of producing pro-inflammatory cytokines and chemokines that cause BBB disruption (57). However, astrocytes are also capable of producing factors that support repair and regeneration after CNS damage (58). At present, the role of astrocytes in TBI is unclear. The removal of proliferating astrocytes in mice has been shown to result in neuronal degeneration and inflammation, and play an essential role in preserving neuronal tissue after moderate (but not severe) TBI (59). In contrast, blocking astrocyte proliferation using agents that disrupt various stages of the cell cycle can lead to reduced neuronal cell death after FPI in rats (60). These actions may depend on the severity, stage, and affected brain area (61). Recent studies also show that astrocytes function as intracranial baroreceptors and play an important role in homeostatic control of arterial blood pressure and brain blood flow (62).

Although our study shows that DC can reduce the accumulation of astrocytes near the trauma site at day 3 and microglia at day 7 after TBI, it is difficult to determine the relationship between gliocytes and the post-TBI prognosis; therefore, more studies are needed to determine the role of gliocytes in DC after TBI.

## Limitation

Traumatic brain injuries can be quite heterogeneous. There are limitations to our experimental design that must be considered before our findings can be translated into the clinical setting. It is difficult for animal models to truly simulate the pathophysiological changes that occur in human clinical situations; there is no perfect animal model that can fully match the changes in human brain trauma. Accordingly, our DC model does not perfectly simulate the clinical features of human brain trauma.

Although this study utilized CCI to ensure that the TBI was standardized and used the same parameters, it does not encompass the full spectrum of TBIs. It is unclear whether DC in other TBI models would produce the same results. In addition, our study groups had limited sizes and comprised only male mice; therefore, the known sex-associated differences in TBI recovery and neuroplasticity could not be addressed. TBI in humans is usually caused by forces striking the frontal, occipital, or temporal areas of the skull. In contrast, TBI in the mouse model is usually induced *via* the skull vertex. A small diameter impactor tip in CCI models produces neither diffuse axonal injury (DAI) nor significant neurological functional deficits, and the CCI mouse model in our study often manifests with a focal injury; it is difficult to simulate diffuse lesions. And the damage to the meninges in CCI models is also not optimal for simulating the pathophysiological changes that occur in human clinical situations.

Functional evaluation of psychological and neurological parameters depends on a patient's verbal communication skills, which cannot be evaluated in mice. Animal experiments lack a scoring system such as the Glasgow Coma Scale (GCS), which evaluates the severity of brain trauma in human TBI patients. In addition, neurological recovery is more rapid and complete in mice. The ICP measurements in our study were performed under anesthesia, which is known to influence cerebrovascular hemodynamics.

Although there is evidence that a sustained elevation in ICP (> 20 mmHg) after severe TBI is associated with a poor outcome, the efficacy of threshold-targeted interventions has not been thoroughly established (16, 63). At the same time, due to the differences in mouse and human species, in our study, the surgical indications after CCI are mostly based on the personal opinions of our researchers, and it is difficult to simulate the surgical indications of DC after CCI in clinical situations.

## Conclusion

In our study, we confirmed that DC and focal debridement can effectively attenuate the increase in ICP and improve cerebral blood flow in mice with severe TBI. Moreover, the removal of contused brain tissue alleviates the neurological dysfunction in the acute stage, reduces the expression of some pro-inflammatory factors [C5/C5a, ICAM-1 (CD54), IL-16, CXCL10 (IP-10/CRG-2), M-CSF, TIMP-1, IL-1 β, and IL-6], reduces neuron apoptosis, diminishes BBB disruption, and regulates the number of glial cells. However, DC and focal debridement negatively affected recovery of neurological function, motor function, and cognition in mice with TBI at intermediate and late stages. Thus, although surgical treatment of patients with TBI can relieve acute symptoms, it may not be conducive to the recovery of neurological function and improving quality of life.

Our mouse model provides an opportunity to systematically evaluate the changes that occur following DC for TBI; however, further research is needed before it can serve as a reference for making clinical decisions.

## Data availability statement

The original contributions presented in the study are included in the article/supplementary material, further inquiries can be directed to the corresponding authors.

## Ethics statement

The animal study was reviewed and approved by Tianjin Medical University General hospital.

## Author contributions

WY and ZW conceived and designed the study. YL, XL, and ZC conducted the experiments and wrote the manuscript. YW, JL, JG, and AH analyzed the data and interpreted the results. All authors contributed to the article and approved the submitted version.

## Funding

This work was supported by the Tianjin Science and Technology Support Key Project (Grant Number: 20YFZCSY00010). The authors are thankful for funding by Tianjin Key Medical Discipline (Specialty) Construction Project.

## Conflict of Interest

The authors declare that the research was conducted in the absence of any commercial or financial relationships that could be construed as a potential conflict of interest.

## Publisher's Note

All claims expressed in this article are solely those of the authors and do not necessarily represent those of their affiliated organizations, or those of the publisher, the editors and the reviewers. Any product that may be evaluated in this article, or claim that may be made by its manufacturer, is not guaranteed or endorsed by the publisher.
